# Evaluation of neurological outcomes after prolonged sedation among ICU patients: A prospective study in India

**DOI:** 10.6026/973206300214072

**Published:** 2025-11-15

**Authors:** Kishore Sekar, Hasan Saleem Manghi, Hadiya Shakil

**Affiliations:** 1 Department of General Surgery, Queens Hospital, London, Romford, United Kingdom; 2 Department of Intensive Care/Accidents and Emergency, Eterna Health, Sindh, Pakistan; 3 Department of Accident & Emergency, Dr. Ziauddin Hospital, Sindh, Pakistan

**Keywords:** Sedation, intensive care unit, neurological outcomes, cognitive recovery, prospective study

## Abstract

Extended sedation in intensive care settings can produce neurological problems. Hence, this prospective study analyzed the neurological recovery of patients receiving continuous sedation (> 72 hours). Neurological function was evaluated at the time of ICU discharge and at three months to assess for any residual cognitive or functional impairment. Most patients appeared to recover completely and achieve their baseline pre-morbid function although a small number had mild persistent cognitive impairment. The data suggest that with continued vigilance after prolonged sedation greater than 72 hours there are generally no significant long-term neurological implications, which can be useful to render safer sedation methods in critical care medicine.

## Background:

The management of prolonged sedation in patients admitted to intensive care units (ICUs) is a key element of supportive care but raises substantial concern for possible neurological sequelae [1]. Sedative medications, when used continuously for long durations, can alter cerebral physiology, impair recovery of arousal and lead to cognitive and functional deficits [2]. Preparation for intensive mechanical ventilation or airway protection often necessitates the purposeful use of sedation, mainly to decrease metabolic requirements and improve patient comfort; however, an inappropriate depth or duration of sedation may prevent neurological recovery or make weaning from mechanical ventilation more complicated [3]. Clinical and experimental studies provide evidence that prolonged sedation is related to longer lasting cognitive deficits, delirium, or poor quality of life, as reported by survivors of critical illness [4]. A large portion of the existing literature, however, is retrospective and/or limited to specific or narrowly defined patient groups. As such, many aspects of long-term neurological trajectories are unclear as they relate to heterogeneous inpatient ICU populations [5]. It is essential to conduct prospective evaluations of neurological function at the time of hospital discharge and at follow-up assessment to better understand the severity and duration of impairments and to assist with the development of sedation protocols that ensure both short-term clinical need and long-term brain health [6]. Therefore, it is of interest to describe the neurological outcomes and recovery trajectories in patients exposed to prolonged sedation in the ICU, both at discharge and at three-month follow-up, in order to inform best practices in sedation management.

## Materials and Methods:

This was a prospective observational study performed in the adult intensive care unit (ICU) of a tertiary care hospital. The study included patients aged 18-80 years, who had the need for continuous sedation for greater than 72 hours. In general, the sedative regimen included propofol and/or midazolam that were administered and titrated based on the sedation protocol in the unit to achieve the desired depth of sedation. The neurological status was assessed at ICU or hospital discharge with standardized cognitive and motor assessment scales. A follow up assessment was completed 3 months after gap, either in a clinic visit or telehealth assessment. Data abstraction included demographic data, sedative duration, cumulative drug dose, comorbidities and complications related to the ICU admission. Statistical analysis utilized descriptive measures along with appropriate comparative tests between patients who had neurological recovery versus patients who continued to have impairment.

## Results: 

Among the 100 ICU patients included, 75% regained full or near-baseline neurological function by the time of discharge, with 85% of these maintaining their recovery at the 3-month follow-up. A quarter of the cohort (25%) exhibited delayed or incomplete recovery at discharge and 10% had persistent mild cognitive impairment at 3 months. Prolonged sedation duration was modestly associated with delayed recovery, though differences in cumulative sedative doses between groups were not statistically significant. Baseline demographics were similar across recovery groups, with hypertension and diabetes being the most common comorbidities. Propofol was the most frequently used sedative, followed by midazolam, with a subset receiving a combination. Neurological recovery outcomes were dynamic, with some patients improving over time while others remained impaired or were lost to follow-up. Statistical comparisons confirmed that longer sedation duration correlated with poorer neurological recovery. ICU complications such as delirium, sepsis and hypoxemia were more frequent in the impaired group, though associations did not reach statistical significance. Importantly, no major adverse neurological events were observed. Collectively, the findings highlight the influence of sedation duration and ICU complications on recovery trajectories among critically ill patients. Table 1 (see PDF) describes the baseline characteristics of the study population, including age, sex, comorbidities and sedative choice. It compares recovered versus impaired groups, showing broadly similar distributions with slightly higher comorbidity rates in the impaired group. Table 2 (see PDF) presents recovery trajectories, showing that most patients maintained or improved their status by 3 months. While some deficits persisted, overall recovery outcomes were favorable. Table 3 (see PDF) compares sedation exposure between recovered and impaired groups. Prolonged sedation duration was significantly linked with delayed recovery, though cumulative sedative doses did not differ significantly. Table 4 (see PDF) lists complications observed during ICU stay and their associations with recovery status. Delirium and sepsis occurred more frequently among impaired patients, though none reached statistical significance.

## Discussion:

This prospective study offers positive evidence that many critically ill patients with prolonged ICU sedation recover neurological function by the time of discharge from hospital and many more maintain this recovery in the following three months [7]. This finding counteracts the historical claim that prolonged sedation always produces poor neurocognitive outcomes and suggests that careful monitoring sedation depth/ duration can enable neurological recovery in the long-term [8]. The finding of a modest relationship between the duration of sedation and delayed recovery (but no relationship between cumulative sedative dose and recovery) provides useful perspectives on the clinical implications of this clinical finding [9]. This distinction suggests the time features of sedation are likely more important than the maximal drug burden in shaping neurological recovery. Protocolized methods of sedation that include daily sedation interruptions, validated sedation scales and individualized depth targets, can be credited with surrounding the risks of neurocognitive impairment and systematic means of applying these methods to sedation may also help to explain the outcomes noted in this cohort [10]. These data positions the notion that although recovery pathways are reasonable from prolonged sedation, cognitive impairment risk still exists in a small fraction of patients. Factors that may contribute to recovery impairment included premorbid brain vulnerability, systemic inflammatory response, hypoxemia, or delirium [11]. Early in the course of recovery, some individuals may have an opportunity for early diagnosis, neurocognitive monitoring and rehabilitation. Viewed from a broader perspective, the data from this study support the precarious and delicate balance of sedating patients in the ICU context [12]. Although sedation is often required for invasive procedures, as well as for comfort during mechanical ventilation, excessive and prolonged sedation may negatively impact neurological recovery. This study indicates that with some attention to dredging the necessary balance with appropriate titration, both short- and possibly long-term preservation of neurological repair could be achieved [13].

Nonetheless, the single cohort design limits generalizability and, while prospective, it did not account for some confounding factors such as delirium burden, severity of illness, or class-specific effects of sedatives. Longer follow-up would be important to determine if the neurological recovery persists beyond three months. Future randomized trials will be needed to replicate our findings and improve standardization of best practices to minimize neurocognitive risk in survivors after an ICU admission. Long-term sedation-based therapy in the ICU, with proper planning and management, seems acceptable for most patients expecting a favorable neurological outcome. Once again, the duration of sedation rather than the total dose seems to be a more appropriate factor by which we can predict recovery from sedative therapy outlining the importance of structured sedation protocols. Although a smaller number of patients may have enduring deficits after very long sedative therapy, on-going assessment and rehabilitation services should be considered. This study adds reliably to the growing body of evidence that sedation practices can be modified to achieve the best balance between clinical need and the preservation of neurological outcome.

## Conclusion: 

The majority of ICU patients receiving extended sedation for medical treatment regained their baseline neurological function at the time of discharge and maintained recovery for three months. Thus, prolonged sedation does not seem to affect long-term neurological outcome in most patients. Persistent cognitive impairment in some patients emphasizes the need for a developed follow-up process and possible rehabilitation that optimizes sedation protocols.
